# Venoarterial extracorporeal membrane oxygenation in the treatment of postinfarction cardiogenic shock: is it the end, or do we need to select patients better?

**DOI:** 10.62675/2965-2774.20240041-en

**Published:** 2024-08-05

**Authors:** Livia Maria Garcia Melro, Marcelo Park, Pedro Vitale Mendes

**Affiliations:** 1 Intensive Care Unit Hospital Samaritano Paulista São Paulo SP Brazil Intensive Care Unit, Hospital Samaritano Paulista - São Paulo (SP), Brazil.; 2 Hospital São Paulo Escola Paulista de Medicina Universidade Federal de São Paulo São Paulo SP Brazil Intensive Care Department, Hospital São Paulo, Escola Paulista de Medicina, Universidade Federal de São Paulo - São Paulo (SP), Brazil.; 3 Hospital das Clínicas Faculdade de Medicina Universidade de São Paulo São Paulo SP Brazil Medical Intensive Care Unit, Hospital das Clínicas, Faculdade de Medicina, Universidade de São Paulo - São Paulo (SP), Brazil.

Despite recent advances in clinical and mechanical support, cardiogenic shock persists with a mortality rate of approximately 50%.^(1)^This challenging scenario motivates an ongoing quest for more effective therapeutic strategies and a deeper understanding through clinical studies to elucidate the role of mechanical support in these patients. Venoarterial extracorporeal membrane oxygenation (VA-ECMO) offers biventricular, cardiopulmonary support^(2)^ and, with the recent publication of randomized trials on the topic, is at the center of debates regarding mechanical support in cardiogenic shock patients.

The Society for Cardiovascular Angiography and Interventions (SCAI) classification is crucial for categorizing cardiogenic shock patients based on the severity of illness in patients who require vasoactive drugs.^(3)^ SCAI C denotes cardiogenic shock requiring inotropic support, SCAI D includes those requiring multiple vasopressors and exhibiting a worsening condition, and SCAI E represents patients in cardiac arrest with severe metabolic acidosis and refractory hypotension. Additionally, the vasoactive inotropic score (VIS) helps measure the severity of cardiogenic shock by indicating the need for vasopressors, providing valuable insights into patient severity and clinical characteristics.^(4)^ The importance of subclassifications in cardiogenic shock arises from the likelihood that only the most severely ill patients should benefit from this type of support, given its association with serious complications such as bleeding, stroke, and sepsis.^(5)^These complications may counterbalance the survival benefits of ECMO.

Until the end of 2022, despite VA-ECMO being a well-established type of mechanical support in Shock Team’s protocols worldwide, guidelines were primarily based on retrospective studies and expert opinions.^(6,7)^ The recommendations lacked the robust foundation of prospective, randomized trials, highlighting the evolving nature of our understanding and the need for continued research to refine guidelines and enhance patient outcomes in cases of cardiogenic shock.

In a pioneering study, the ECMO-CS (Extracorporeal Membrane Oxygenation in the Therapy of Cardiogenic Shock) trial randomized 122 patients with acute myocardial infarction (AMI) complicated by cardiogenic shock.^(8)^ The trial compared the immediate initiation of ECMO to an initial conservative management approach, with the provision for ECMO rescue in case of continued clinical deterioration. Despite the broad inclusion criteria, which included patients who fell under SCAI categories C, D and E, the initial characteristics of the participants suggested a cohort with a reasonably severe condition. These patients presented with high doses of vasopressors, as evidenced by a VIS of approximately 60, along with elevated lactate levels of approximately 5mmol/L. Although the results indicated no significant difference in mortality between the groups, the notable occurrence of approximately 40% crossover from conventional treatment to ECMO introduces complexity to the interpretation. This observation raises questions about the optimal timing for ECMO as a rescue strategy but emphasizes the feasibility of an initial conservative approach and the escalation of support with ECMO in cases of clinical deterioration. The randomization of patients from different SCAI categories further underscores the need for additional research to explore the nuanced application of VA ECMO across varying degrees of cardiogenic shock severity.

A subsequent study, the ECLS (Extracorporeal Life Support) Shock Trial, enrolled 420 post-AMI cardiogenic shock patients randomized to ECMO or conventional clinical management.^(1)^ Once again, no difference in mortality was observed between the groups. However, interpreting the results becomes more complex considering that 50% of patients were SCAI C shock category, characterized by lower severity and a lower likelihood of receiving ECMO in real-world scenarios. Furthermore, in the control group, 12.5% of patients received ECMO, and another 15.4% received other types of mechanical support, making the interpretation of the results challenging.

In the ECLS-shock study, the lower severity was reflected in an overall mortality rate of 50%, as well as in cases where the support duration was less than 3 days. Noteworthy characteristics indicating lower severity in the ECLS-shock study randomization included a heart rate of less than 100bpm and a systolic blood pressure exceeding 90mmHg, although they had elevated lactate levels (median of 6.8mmol/L), and 78% of participants had experienced cardiac arrest with a median duration of 20 minutes. This study also revealed a low incidence of left ventricular venting, and the left ventricular decompression in these scenarios appeared to be associated with better outcomes, especially in post–cardiac arrest patients, even though a specific protocol was followed to determine the need for decompression.^(9,10)^ These nuances highlight the importance of considering various factors that may impact patient outcomes when interpreting the results of this study.

Additional factors were found to contribute to this analysis. The ECLS-shock study lacked detailed information on vasopressor doses, while ECMO-CS revealed a median VIS of 60. Both studies share a median subject age of above 60 years, and considering that older age is associated with a worse prognosis, questions persist about the applicability of these results to a younger population.^(11)^

**Figure 1:**
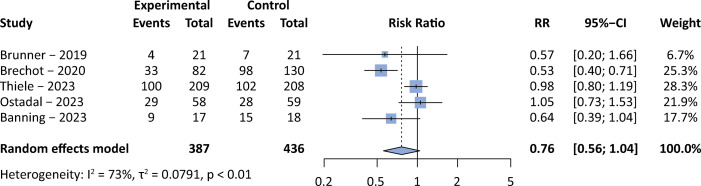
Pooled weighted mortality analysis of selected studies in cardiogenic shock patients allowing the evaluation of venoarterial extracorporeal membrane oxygenation support in comparison to conventional treatment. The Mantel-Haenszel method was used to compute individual and pooled risk ratios. The R-free source software v3.2.5 and meta package were used for building the analyses and graphs. RR - risk ratio; 95%CI - 95% confidence interval.

Despite their limitations, these are the main studies available in the current literature. Smaller studies or those with lower methodological quality still showed conflicting results. Here, we present the results of a pooled weighted mortality analysis including randomized trials and a unique cohort with a matching technique evaluating the use of VA-ECMO support in patients with cardiogenic shock.^(1,8,12-14)^Figure 1 shows the pooled analysis of the data indicating a possible survival benefit of VA-ECMO. The use of therapeutic failure, rather than mortality, as the primary outcome of the meta-analysis may have produced more favorable results for the use of VA-ECMO in patients with cardiogenic shock. On the other hand, if only randomized trials were included, the results would be definitely neutral.

An observational study by Na et al. demonstrated that ECMO might have a positive impact on reducing mortality, particularly in extremely severe cases with a VIS exceeding 130.^(15)^ Importantly, in patients with a VIS below 85, conventional treatment appears to be more beneficial. This distinction is crucial because patients who reach such high vasopressor requirements face a mortality rate exceeding 70%, which contrasts with the 49% observed in the control group of the ECLS shock study. It is possible that in cardiogenic shock, one size does not fit all, and using the VIS can help to identify which patients have the greatest benefit from the use of VA-ECMO. The side effects of VA-ECMO may simply not be worth in a less severe population.

In the context of Brazil being a low-middle-income country, the decision to initiate ECMO presents an even greater challenge due to considerations of limited technological resources. The financing of such advanced support in Brazil remains deficient, as private health insurance may not fully cover the costs, given that the therapy is categorized by the National Agency of Supplementary Health (*Agência Nacional de Saúde Suplementar* - ANS) as extracorporeal circulation via thoracotomy (central cannulation ECMO). This lack of coverage by the Brazilian Unified Health System (*Sistema Único de Saúde* - SUS) further exacerbates health care inequality, as ECMO is primarily provided in private health care services. As outlined in the scientific-technical report submitted for *Comissão Nacional de Incorporação de Tecnologias no Sistema Único de Saúde* (CONITEC) evaluation by our group, another significant limitation involves the training and accreditation of centers throughout Brazil.^(16)^ Ensuring that these centers have the training and expertise to handle complex patients who require extensive resources adds an additional layer of complexity to the decision-making process surrounding ECMO implementation.

Given these challenges and uncertainties, the use of VA-ECMO in post–acute myocardial infarction cardiogenic shock should not be considered a routine strategy. Until further studies emerge, its use is recommended for specific cases, such as those involving younger patients with high vasopressor needs (SCAI D/E), who are treated with a rescue strategy in the presence of clinical deterioration. The ongoing quest for answers, coupled with a judicious approach, is essential to guide clinical decisions and advance the treatment of this complex medical challenge.
