# Supplementary material for: Venoarterial extracorporeal membrane oxygenation in the treatment of postinfarction cardiogenic shock: is it the end, or do we need to select patients better?
Source: Crit Care Sci. 2024 Aug 5;36:e20240041en. doi: 10.62675/2965-2774.20240041-en (PMC11321715; doi:10.62675/2965-2774.20240041-en)
Supplement: Translation

# Translation: Oxigenação por membrana extracorpórea venoarterial no tratamento do choque cardiogênico secundário a infarto: é o fim ou precisamos selecionar melhor os pacientes?

**DOI:** 10.62675/2965-2774.20240041-pt

**Authors:** Livia Maria Garcia Melro, Marcelo Park, Pedro Vitale Mendes

**Affiliations:** 1 Brasil Unidade de Terapia Intensiva, Hospital Samaritano Paulista - São Paulo (SP), Brasil.; 2 Brasil Departamento de Medicina Intensiva, Hospital São Paulo, Escola Paulista de Medicina, Universidade Federal de São Paulo - São Paulo (SP), Brasil; 3 Brasil Unidade de Terapia Intensiva Clínica, Hospital das Clínicas, Faculdade de Medicina, Universidade de São Paulo - São Paulo (SP), Brasil.

Apesar dos recentes avanços no suporte clínico e mecânico, o choque cardiogênico persiste com uma taxa de mortalidade de aproximadamente 50%.^(1)^Esse cenário desafiador motiva uma busca contínua por estratégias terapêuticas mais eficazes e uma compreensão mais profunda por meio de estudos clínicos para elucidar o papel do suporte mecânico nesses pacientes. A oxigenação por membrana extracorpórea venoarterial (ECMO-VA) oferece suporte cardiopulmonar biventricular^(2)^ e, com a publicação recente de estudos randomizados sobre o assunto, está no centro dos debates sobre o suporte mecânico em pacientes com choque cardiogênico.

A classificação da *Society for Cardiovascular Angiography and Interventions* (SCAI) é fundamental para categorizar os pacientes com choque cardiogênico segundo a gravidade da doença em pacientes que necessitam de drogas vasoativas.^(3)^ Uma classificação SCAI C indica choque cardiogênico que requer suporte inotrópico; SCAI D inclui aqueles que requerem vários vasopressores e apresentam piora da condição; e a SCAI E representa pacientes em parada cardiorrespiratória, com acidose metabólica grave e hipotensão refratária. Em conjunto com a classificação da SCAI, o escore inotrópico vasoativo (VIS - *vasoactive inotropic score*) ajuda a medir a gravidade do choque cardiogênico, ao indicar a necessidade de vasopressores, fornecendo informações valiosas sobre a gravidade do paciente e as características clínicas.^(4)^ A importância das subclassificações no choque cardiogênico decorre da hipótese de que apenas os pacientes mais graves se beneficiem desse tipo de suporte, dada sua associação com complicações graves, tais como sangramento, acidente vascular cerebral e sepse.^(5)^ Assim, essas possíveis complicações podem contrabalançar os potenciais benefícios de sobrevida com o uso da ECMO.

Até o fim de 2022, apesar de a ECMO-VA ser uma estratégia bem estabelecida de suporte mecânico nos protocolos de choque em todo o mundo, as diretrizes baseavam-se principalmente em estudos retrospectivos e opiniões de especialistas.^(6,7)^ As recomendações não eram embasadas em estudos prospectivos e randomizados, destacando a necessidade de pesquisas para refinar as diretrizes e melhorar os resultados dos pacientes em casos de choque cardiogênico.

O estudo pioneiro ECMO-CS (*Extracorporeal Membrane Oxygenation in the Therapy of Cardiogenic Shock*) randomizou 122 pacientes com infarto agudo do miocárdio (IAM) complicado por choque cardiogênico.^(8)^ O estudo comparou o início imediato da ECMO a uma abordagem inicial de tratamento conservador, com a previsão de resgate da ECMO em caso de deterioração clínica. Apesar dos amplos critérios de inclusão, que incluíam pacientes que se enquadravam nas categorias C, D e E da SCAI, as características iniciais dos participantes sugeriam uma coorte com uma condição bastante grave. Esses pacientes apresentavam altas doses de vasopressores, conforme evidenciado por um VIS de aproximadamente 60, com níveis de lactato de aproximadamente 5mmol/L. Além disso, embora os resultados não tenham indicado diferença significativa na mortalidade entre os grupos, a ocorrência de aproximadamente 40% de transição do grupo de tratamento convencional para o grupo ECMO introduz complexidade na interpretação dos resultados do estudo. Essa observação levanta questões sobre o momento ideal em que a ECMO deve ser usada como estratégia de resgate. No entanto, enfatiza a viabilidade de uma abordagem que consiste em uma estratégia conservadora inicial e o aumento do suporte com o uso de ECMO em casos de deterioração clínica. A randomização de pacientes de diferentes categorias da SCAI ressalta ainda mais a necessidade de novas pesquisas para explorar a aplicação da ECMO-VA em diferentes graus de gravidade do choque cardiogênico.

Posteriormente, o estudo ECLS (*Extracorporeal Life Support*) *Shock Trial*, incluiu 420 pacientes com choque cardiogênico secundário ao IAM randomizados para ECMO ou tratamento clínico convencional.^(1)^ Mais uma vez, não foi observada diferença na mortalidade entre os grupos. No entanto, a interpretação dos resultados torna-se mais complexa, considerando que 50% dos pacientes pertenciam à categoria de choque SCAI C, caracterizada por menor gravidade e menor probabilidade de receber ECMO em cenários concretos. Além disso, no grupo controle, 12,5% dos pacientes receberam ECMO, e outros 15,4% receberam outros tipos de suporte mecânico, o que torna a interpretação dos resultados desafiadora.

No ECLS, a menor gravidade dos pacientes incluídos é percebida pela taxa de mortalidade geral de 50% e uma duração do suporte inferior a 3 dias. Além disso, outras características dignas de nota que indicam uma menor gravidade na randomização do estudo ECLS incluíram frequência cardíaca <100bpm e pressão arterial sistólica superior a 90mmHg, embora os tivessem níveis elevados de lactato (mediana de 6,8mmol/L) e 78% dos participantes tivessem sofrido parada cardiorrespiratória com duração mediana de 20 minutos. Por fim, no estudo ECLS houve um baixo uso de estratégias de descompressão do ventrículo esquerdo, estratégia essa que parece estar associada a desfechos mais favoráveis, especialmente em pacientes vítimas de parada cardiorrespiratória.^(9,10)^ Assim, essas nuances destacam a importância de considerar vários fatores que podem afetar os desfechos de pacientes ao interpretar os resultados desses estudos.

Outros fatores que devem ser considerados na análise de ambos os trabalhos incluem: o ECLS não tinha informações detalhadas sobre as doses de vasopressores, enquanto o ECMO-CS tinha pacientes com um escore VIS mediano de 60. Além disso, os pacientes de ambos os estudos tinham uma idade mediana acima de 60 anos e, considerando que a idade avançada está associada a um prognóstico mais desfavorável, persistem dúvidas sobre a aplicabilidade dos resultados obtidos em uma população mais jovem.^(11)^

No entanto, apesar de suas limitações, esses são os principais estudos disponíveis na literatura atual, tendo em vista que estudos menores ou com qualidade metodológica inferior apresentaram resultados conflitantes. Assim, com o objetivo de compilar o que há de dado atualmente disponível, apresentamos aqui os resultados de uma análise de mortalidade ponderada combinada que inclui os estudos randomizados e uma coorte única de pacientes que avalia o uso do suporte com ECMO-VA em pacientes com choque cardiogênico.^(1,8,12-14)^A figura 1 mostra a análise conjunta dos dados que indica um possível benefício de sobrevida com o suporte com ECMO-VA. O uso de falha terapêutica, em vez de mortalidade, como desfecho primário da metanálise pode ter produzido resultados mais favoráveis ao uso de ECMO-VA em pacientes com choque cardiogênico. Por outro lado, se apenas os estudos randomizados fossem incluídos, os resultados seriam definitivamente neutros.

**Figura 1:** Análise de mortalidade ponderada combinada de estudos selecionados com pacientes em choque cardiogênico, permitindo a avaliação do suporte de oxigenação por membrana extracorpórea venoarterial em comparação com o tratamento convencional. Utilizou-se o método de Mantel-Haenszel para calcular as razões de risco individuais e combinadas. O *software* de fonte livre R v3.2.5 e o pacote meta foram usados para construir as análises e os gráficos. RR - razão de risco; IC95% - intervalo de confiança de 95%.

Na et al., em estudo observacional, demonstraram que a ECMO pode ter um efeito positivo na redução da mortalidade, principalmente em casos extremamente graves com VIS > 130.^(15)^ É importante ressaltar que, em pacientes com VIS < 85, o tratamento convencional parece ser mais benéfico. Essa distinção é crucial, pois os pacientes que atingem altas doses de vasopressores apresentam taxa de mortalidade superior a 70%, o que contrasta com os 49% observados no grupo controle do estudo ECLS. É possível que, no choque cardiogênico, uma única estratégia não sirva para todos, e o uso do VIS pode ajudar a identificar quais pacientes são mais graves e, assim, se beneficiam mais do suporte com ECMO-VA. Em suma, os efeitos colaterais da ECMO-VA podem simplesmente superar os potenciais benefícios em uma população menos grave.

No contexto do Brasil, que é um país de baixa e média renda, a decisão de iniciar a ECMO representa desafio ainda maior devido à limitação dos recursos tecnológicos. O financiamento desse suporte avançado no Brasil continua deficiente, pois os planos de saúde privados podem não cobrir totalmente os custos, uma vez que o tratamento é classificado pela Agência Nacional de Saúde Suplementar (ANS) como circulação extracorpórea via toracotomia (ECMO de canulação central). Essa falta de cobertura pelo Sistema Único de Saúde (SUS) agrava ainda mais a inequidade na assistência médica, uma vez que a ECMO é fornecida principalmente em serviços de saúde privados. Conforme descrito no relatório técnico-científico apresentado para avaliação da Comissão Nacional de Incorporação de Tecnologias (CONITEC) no SUS por nosso grupo, outra limitação significativa envolve o treinamento e o credenciamento de centros em todo o Brasil.^(16)^ Garantir que esses centros tenham o treinamento e a especialização para lidar com pacientes complexos e que exigem grande quantidade de recursos confere maior complexidade ao processo de tomada de decisão em torno da implementação da ECMO.

Assim, em virtude desses desafios e incertezas, o uso da ECMO-VA no choque cardiogênico secundário ao IAM não deve ser considerado uma estratégia de rotina. À espera de mais estudos, o uso da ECMO-VA segue recomendado para casos específicos, em pacientes mais jovens, com alta necessidade de vasopressores (SCAI D/E), e que demandam uma estratégia de resgate por deterioração apesar de suporte clínico otimizado. A busca contínua por respostas, aliada a uma abordagem criteriosa na seleção dos pacientes, é essencial para orientar as decisões clínicas e avançar no tratamento desse complexo desafio médico.
